# Comparative Evaluation of Pericervical Dentin Preservation and Fracture Resistance of Root Canal-Treated Teeth Prepared Using Instrumentation Systems With Different Tapers: An In Vitro Study

**DOI:** 10.7759/cureus.117731

**Published:** 2026-10-10

**Authors:** Khushbu B Joshi, Jitendra Lohar, Prerna Thareja, Rajvika Sharma, Komal Pareek, Amardeep Tanwar

**Affiliations:** 1 Department of Conservative Dentistry and Endodontics, Pacific Dental College and Research Centre, Udaipur, IND; 2 Department of Oral Medicine and Radiology, Pacific Dental College and Research Centre, Udaipur, IND

**Keywords:** cone-beam computed tomography, file taper, fracture resistance, mandibular premolars, pericervical dentin, root canal preparation, rotary files

## Abstract

Background and objective

Pericervical dentin (PCD) preservation is a crucial consideration in modern endodontics, as excessive removal of dentin during root canal preparation could jeopardize the structural strength of endodontically treated teeth. The objective of this in vitro study was to compare PCD preservation and fracture resistance among various instrumentation systems with different tapers in root canal-treated mandibular premolars.

Materials and methods

Forty freshly extracted, intact, single-rooted mandibular first premolars were randomly selected and subdivided into four groups (n = 10). Groups I, II, III, and IV were prepared using stainless steel hand files with a 2% taper, TruNatomy rotary files with a 4% taper (Dentsply Sirona, Ballaigues, Switzerland), ProTaper Gold files up to F2 (Dentsply Sirona, Charlotte, United States), and HyFlex rotary files with a 6% taper (Coltène/Whaledent, Langenau, Germany), respectively. The pre- and post-instrumentation thickness of the PCD was assessed at the cementoenamel junction (CEJ) using cone-beam computed tomography (CBCT). After instrumentation, the canals were obturated, and the teeth were restored with composite resin. Statistical analysis was carried out using the paired Student's t-test, one-way ANOVA, and Tukey's post hoc test, with statistical significance set at p < 0.05. Fracture resistance was assessed using a universal testing machine at a crosshead speed of 1 mm/min.

Results

A statistically significant reduction in PCD thickness was observed following instrumentation in all four groups. The mean PCD loss was 0.35 ± 0.10 mm for hand files, 0.23 ± 0.08 mm for TruNatomy, 0.28 ± 0.11 mm for ProTaper Gold, and 0.42 ± 0.09 mm for HyFlex. One-way ANOVA demonstrated a statistically significant difference in PCD reduction among the four instrumentation groups (F(3,36) = 7.50, p = 0.001). Fracture resistance was highest in the ProTaper Gold group (724.91 ± 149.38 N), followed by TruNatomy (706.73 ± 134.52 N), HyFlex (654.68 ± 139.76 N), and hand files (635.42 ± 59.87 N).

Conclusion

All four instrumentation systems produced measurable reductions in PCD thickness following root canal preparation. TruNatomy demonstrated the lowest numerical PCD reduction, whereas HyFlex demonstrated the highest numerical reduction. ProTaper Gold showed the highest numerical fracture resistance, followed by TruNatomy, HyFlex, and hand files. The observed numerical differences should be interpreted according to the final validated statistical analysis rather than as evidence of superiority between instrumentation systems.

## Introduction

The goal of endodontics is to remove microorganisms from the root canal system and maintain the natural dentition in terms of function and esthetics. It is not only necessary to thoroughly clean and shape the root canal system during root canal therapy but also to preserve the remaining sound dentinal structure [1]. Excessive removal of radicular dentin during access preparation and canal instrumentation may adversely affect the structural integrity of endodontically treated teeth and increase their susceptibility to fracture [2]. In recent years, the principles of minimal intervention in endodontics have been increasingly emphasized to preserve as much dentinal structure as possible while still achieving adequate debridement of the canals [3].

Pericervical dentin (PCD) has been reported to play an important role in the biomechanical behavior of endodontically treated teeth and is generally described as the dentin that extends approximately 4 mm above and below the crestal bone. This region is important for stress distribution and fracture resistance, and its preservation is considered particularly relevant [4]. Experimental research has also shown that excessive instrumentation of the canals can be detrimental to the fracture resistance of the treated tooth, depending on the amount of dentin removed, the instrumentation system used, and the experimental methods employed [5].

The introduction of nickel-titanium (NiTi) rotary instruments has significantly reduced the shortcomings of conventional stainless steel (S/S) instruments in terms of flexibility, cutting efficiency, and preservation of the original root canal anatomy [6]. The amount of dentin removed during preparation, however, depends on the design, taper, size, motion, and metallurgical properties of the instrument [7]. A higher-taper rotary system might remove more radicular dentin, especially from the cervical and middle thirds of the root, which could compromise the mechanical integrity of the remaining tooth structure [8]. Hence, systems with minimal taper and anatomically conservative instrumentation have been developed to minimize unnecessary dentin removal while still allowing effective root canal shaping [9].

The concept of minimally invasive endodontics has, therefore, attracted considerable interest. Conservative access preparation and reduced-taper instrumentation aim to preserve tooth structure while achieving the biological goals of root canal therapy [10]. However, there is conflicting evidence regarding the association between conservative instrumentation and fracture resistance. In maxillary premolars, for instance, there was no statistically significant difference in fracture resistance between preparations with 4% and 6% tapers, while other studies revealed that smaller-taper systems produced greater fracture resistance after instrumentation [11]. These differences suggest that a variety of interacting factors, such as remaining dentin thickness, canal anatomy, access preparation, instrumentation design, and restorative procedures, may play a role in fracture resistance [12].

Fracture resistance is one of the main factors used to determine the biomechanical properties of endodontically treated teeth. Structural changes in radicular dentin may occur during root canal preparation and may include dentinal defects and microcracks. In a previous microscopic investigation, some rotary systems were thought to promote the formation of dentinal microcracks after instrumentation [13]. Some subsequent studies, however, have cast doubt on whether instrumentation necessarily causes new defects, especially with modern heat-treated NiTi systems [8,9]. The findings of these studies highlight the need to evaluate the amount of dentin removed, as well as the impact of specific file designs and instrumentation kinematics on radicular dentin [14].

New developments in NiTi metallurgy, especially the thermomechanically treated and the heat-treated alloys, have led to instruments being produced with increased flexibility and decreased cyclic fatigue [15]. These characteristics allow for more controlled canal preparation and may facilitate conservative shaping protocols. However, findings regarding dentinal defect formation following instrumentation with heat-treated systems are inconsistent across studies. Previous investigations have indicated that thermally treated systems exhibit no extra microcracks when tested using micro-computed tomography, while some other investigations reveal differences in crack incidence among the different systems [16,17]. Therefore, the biomechanical properties of contemporary NiTi instruments must be considered alongside their taper and shaping characteristics.

Knowledge of the relationship between file taper, dentin preservation, and fracture resistance can help clinicians select appropriate instrumentation protocols that balance adequate canal preparation with maintaining the structural integrity of endodontically treated teeth.

## Materials and methods

The present in vitro study was conducted in the Department of Conservative Dentistry and Endodontics, Pacific Dental College and Research Centre, Udaipur, Rajasthan.

Forty freshly extracted, intact, single-rooted mandibular first premolars extracted for orthodontic purposes were included. Teeth with fractures, cracks, caries, previous endodontic treatment, root resorption, developmental anomalies, or abnormal root length were excluded (Figure 1).

**Figure 1:**
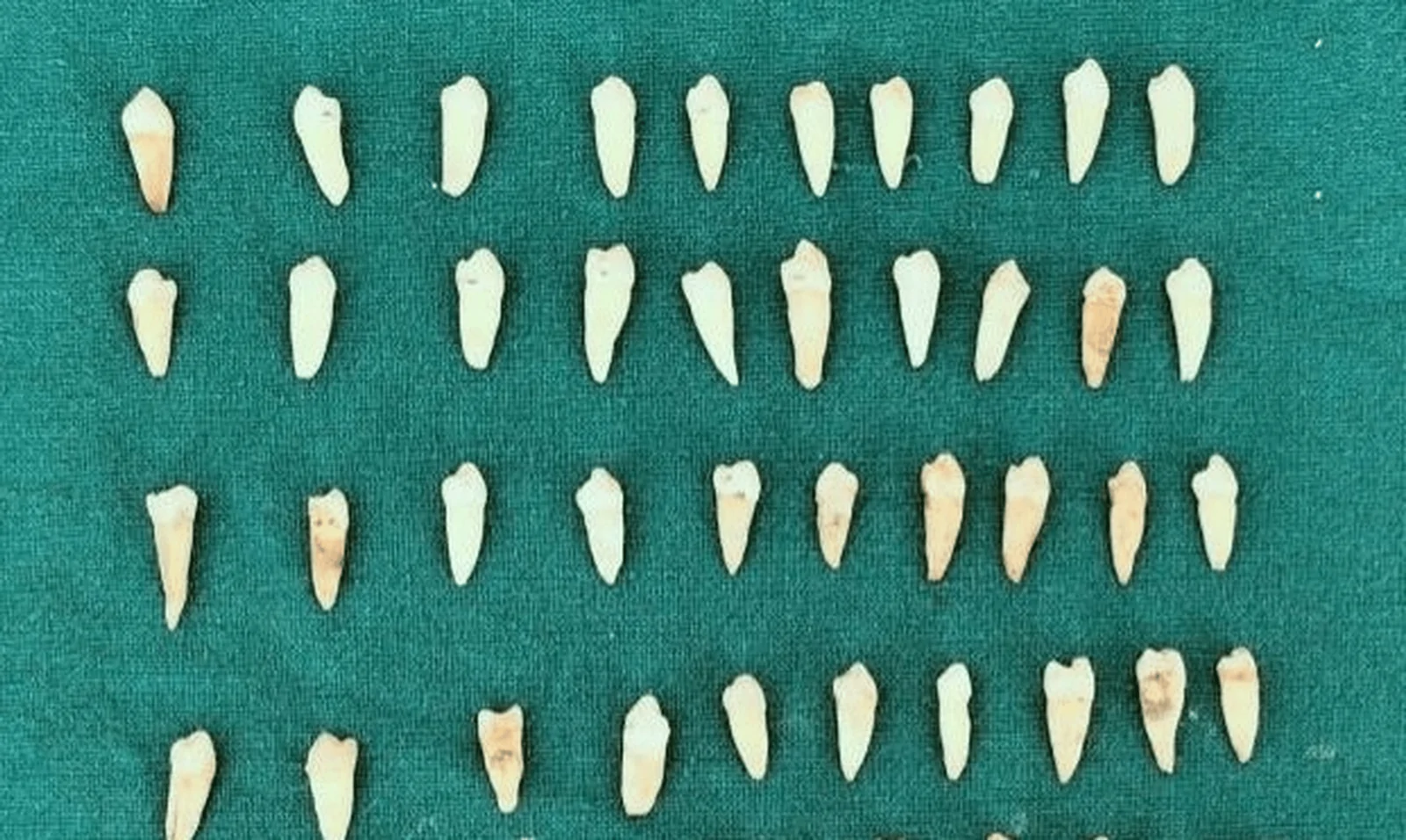
Selection of forty freshly extracted, intact, single-rooted mandibular first premolars for the in vitro study. Forty freshly extracted mandibular first premolars meeting the predefined inclusion criteria were selected, cleaned, and prepared for allocation into four instrumentation groups.

Selection criteria for samples

Forty freshly extracted, intact, single-rooted mandibular first premolars were selected for the study. The teeth had been extracted for orthodontic purposes and were considered suitable for mounting and cone-beam computed tomography (CBCT) analysis.

Teeth were excluded if they exhibited abnormal root length, structural damage such as fractures or cracks, previous endodontic treatment, root resorption, developmental anomalies, or caries.

Root canal preparation

Following sample selection, access cavities were prepared to establish straight-line access to the root canals. The sample size was determined using an a priori power analysis for a one-way ANOVA involving four independent groups. Considering an effect size (Cohen's f) of 0.50, a significance level of 0.05, and a statistical power of 80%, the estimated sample size was approximately 40 specimens, with 10 specimens allocated to each group. Accordingly, 40 freshly extracted, intact, single-rooted mandibular first premolars were selected and randomly divided into four experimental groups (n = 10 each).

Group I 

Root canals were prepared using S/S hand K-files with a 2% taper, sequentially up to ISO size 30.

Group II 

Root canals were prepared using the TruNatomy rotary instrumentation system (Dentsply Sirona, Ballaigues, Switzerland) with a 4% taper at a rotational speed of 500 rpm and a torque of 1.5 Ncm, following the manufacturer's recommended instrumentation protocol.

Group III 

Root canal preparation was performed using the ProTaper Gold system (Dentsply Sirona, Charlotte, United States) in the sequence SX, S1, S2, F1, and F2, using an in-and-out motion.

Group IV 

Root canals were prepared using the HyFlex rotary instrumentation system (Coltène/Whaledent, Langenau, Germany) according to the manufacturer's recommended sequence.

During instrumentation, 5.25% sodium hypochlorite was used as an irrigant, and EDTA was used as a lubricant. Recapitulation was performed using an ISO size 10 K-file.

Cleaning and shaping were performed by two calibrated researchers using the specified instrumentation protocols. To minimize operator-related variability, all specimens were prepared according to a standardized protocol. Measurements were recorded using a calibrated measuring system by an examiner who was blinded to group allocation.

Assessment of PCD

Following completion of root canal instrumentation, CBCT imaging was performed for each specimen. The thickness of the PCD was evaluated at the level of the cementoenamel junction (CEJ) using the pre- and post-instrumentation CBCT images. The change in PCD thickness was subsequently calculated.

The pre- and post-instrumentation CBCT images used for assessment are shown in Figure 2.

**Figure 2:**
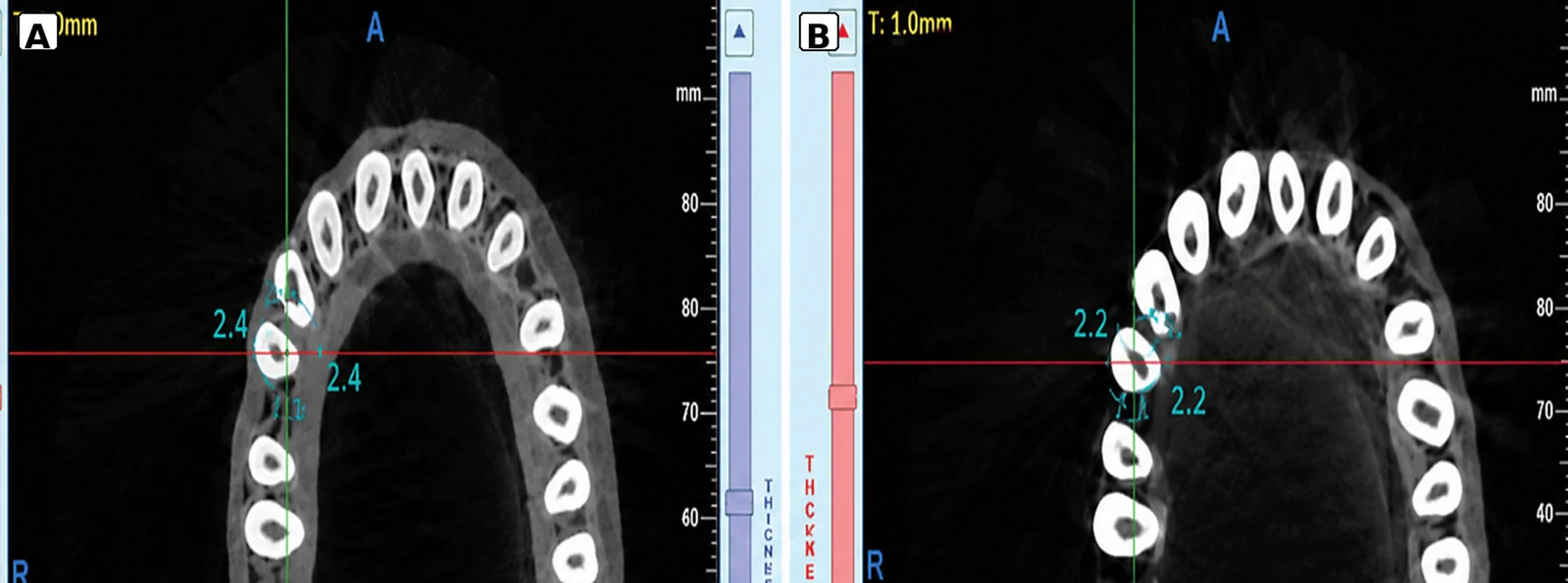
Pre- and post-instrumentation CBCT assessment of pericervical dentin. Representative CBCT images demonstrating the measurement of pericervical dentin thickness at the cementoenamel junction before and after root canal instrumentation. CBCT: Cone-beam computed tomography.

Obturation and coronal restoration

Following instrumentation, the canals were dried using paper points. Single-cone obturation was performed using gutta-percha and AH Plus sealer (Dentsply Sirona, York, United States) in all groups except Group I. In Group I, obturation was performed using the lateral condensation technique. Excess obturation material was removed from the pulp chamber, and the access cavities were restored with composite resin.

The prepared specimens and their mounting arrangement are shown in Figure 3. The instrumentation procedures used for the different groups are shown in Figure 4.

**Figure 3:**
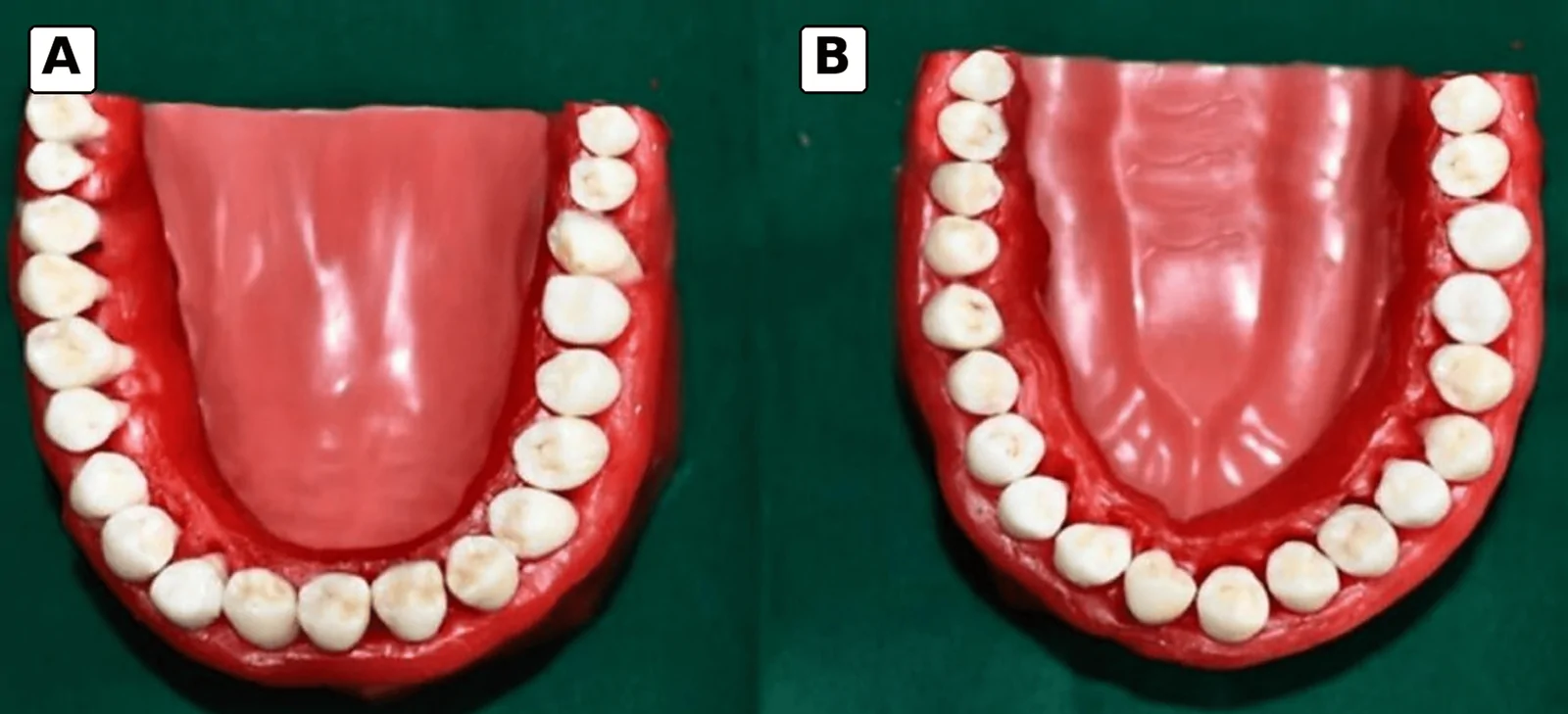
Mounting of prepared specimens on a typodont for standardized testing. Representative specimens positioned in a typodont before completion of the experimental procedures and fracture resistance assessment.

**Figure 4:**
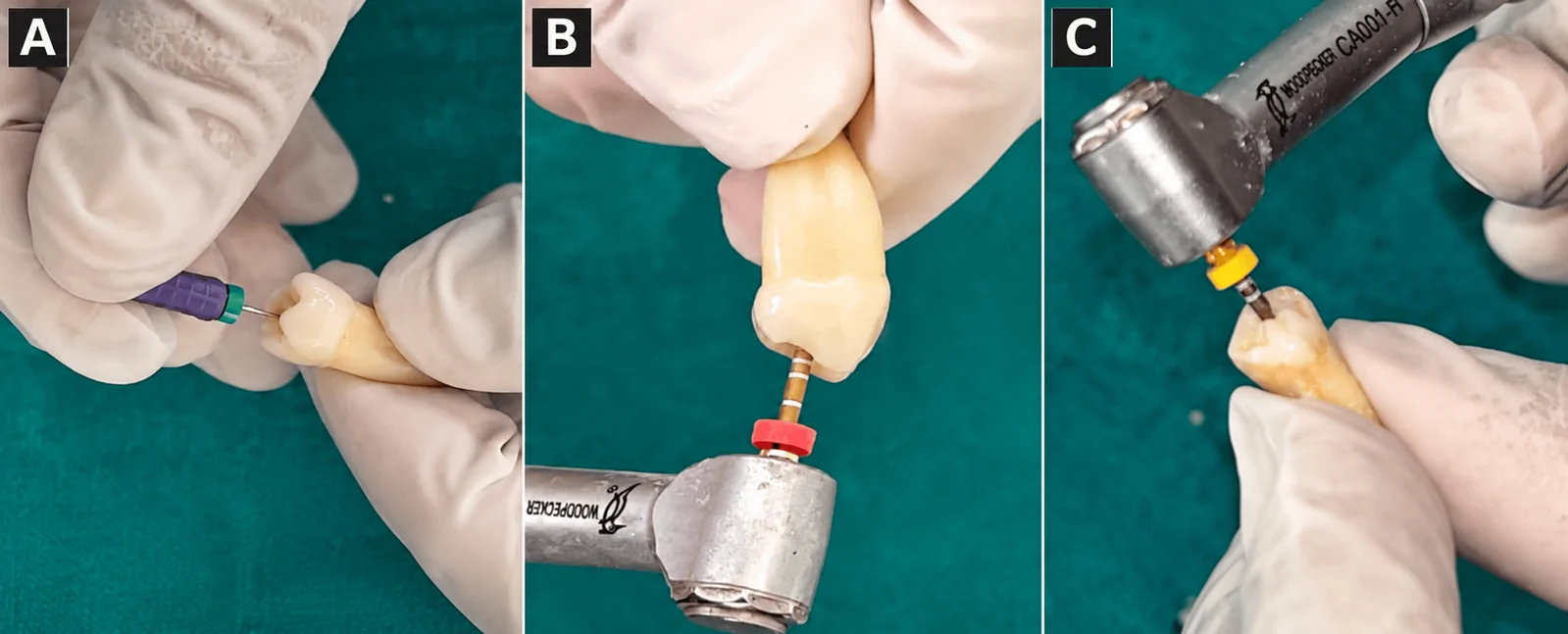
Root canal preparation and obturation procedures used in the experimental groups. (A) Root canal preparation using the ProTaper Gold instrumentation system; (B) single-cone obturation performed in the rotary instrumentation groups; and (C) lateral condensation obturation performed in the hand-file group.

Fracture resistance assessment

The fracture resistance of the prepared specimens was evaluated using a universal testing machine manufactured by FIE (Fuel Instruments & Engineers Pvt. Ltd., Ichalkaranji, Maharashtra, India). Each specimen was mounted in a holder attached to the lower arm of the universal testing machine. A metal indenter was attached to the upper arm and positioned to apply an increasing load to the specimen until fracture occurred.

The crosshead speed was set at 1.0 mm/min, and the load was applied along the long axis of the tooth. The load at failure was recorded in newtons (N) and was considered the primary outcome for fracture resistance assessment. A representative fractured tooth following fracture resistance testing is shown in Figure 5.

**Figure 5:**
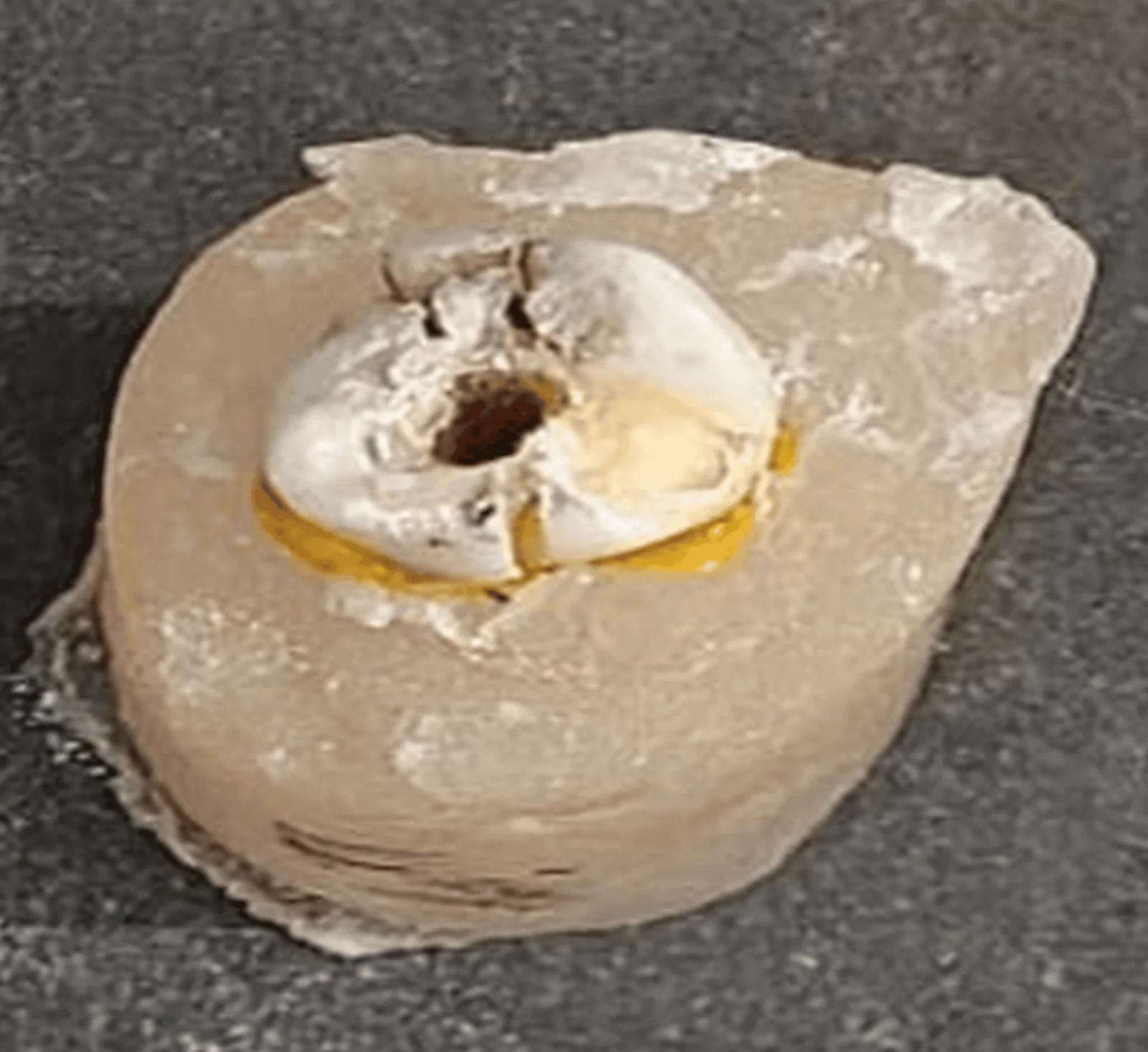
Representative fractured tooth following fracture resistance testing. Representative specimen demonstrating a fracture following the application of an increasing axial load using a universal testing machine.

Statistical analysis

The data were analyzed using IBM SPSS Statistics version 24.0 (IBM Corporation, Armonk, New York, United States). Continuous variables were expressed as mean ± SD. The Shapiro-Wilk test was used to assess normality. As the data were reported to follow a normal distribution, parametric tests were applied. Paired Student's t-tests were used to compare pre- and post-instrumentation PCD thickness within each group. One-way ANOVA was used to compare PCD thickness reduction and fracture resistance among the four instrumentation groups, followed by Tukey's post hoc test for pairwise comparisons. A p-value < 0.05 was considered statistically significant.

## Results

PCD thickness

A total of 40 mandibular first premolars were included and equally allocated among the four instrumentation groups, with 10 specimens in each group. The pre- and post-instrumentation PCD thickness measurements are presented in Table 1. The mean pre-instrumentation PCD thickness was 2.45 ± 0.20 mm in Group I, 2.48 ± 0.22 mm in Group II, 2.50 ± 0.25 mm in Group III, and 2.47 ± 0.23 mm in Group IV. Following instrumentation, the corresponding mean PCD thicknesses were 2.10 ± 0.18 mm, 2.25 ± 0.20 mm, 2.22 ± 0.21 mm, and 2.05 ± 0.19 mm, respectively.

**Table 1:** Descriptive statistics of pre- and post-instrumentation pericervical dentin thickness. PCD: Pericervical dentin.

Group	File system	Pre-instrumentation PCD thickness, mean ± SD (mm)	Post-instrumentation PCD thickness, mean ± SD (mm)	Mean reduction (mm)	PCD loss (%)
I	Hand files (2%)	2.45 ± 0.20	2.10 ± 0.18	0.35	14.29
II	TruNatomy (4%)	2.48 ± 0.22	2.25 ± 0.20	0.23	9.27
III	ProTaper Gold (F2)	2.50 ± 0.25	2.22 ± 0.21	0.28	11.2
IV	HyFlex rotary (6%)	2.47 ± 0.23	2.05 ± 0.19	0.42	17

The mean reductions in PCD thickness were 0.35 ± 0.10 mm in Group I, 0.23 ± 0.08 mm in Group II, 0.28 ± 0.11 mm in Group III, and 0.42 ± 0.09 mm in Group IV. The corresponding percentage reductions were 14.29%, 9.27%, 11.20%, and 17.00%, respectively. The smallest numerical reduction was observed in the TruNatomy group, whereas the largest was observed in the HyFlex group (Table 1).

Intragroup Comparison of PCD Thickness

The intragroup comparison of pre- and post-instrumentation PCD thickness is presented in Table 2. A statistically significant reduction in PCD thickness was observed following instrumentation in all four groups. Group I showed a reduction from 2.45 ± 0.20 mm to 2.10 ± 0.18 mm (t = 5.21, p = 0.001). Group II showed a reduction from 2.48 ± 0.22 mm to 2.25 ± 0.20 mm (t = 4.18, p = 0.002). Group III showed a reduction from 2.50 ± 0.25 mm to 2.22 ± 0.21 mm (t = 5.89, p = 0.001), while Group IV showed a reduction from 2.47 ± 0.23 mm to 2.05 ± 0.19 mm (t = 4.61, p < 0.001).

**Table 2:** Intragroup comparison of pre- and post-instrumentation pericervical dentin thickness. PCD: Pericervical dentin.

Group	Pre-instrumentation PCD thickness, mean ± SD (mm)	Post-instrumentation PCD thickness, mean ± SD (mm)	t-value	p-value	Significance
I	2.45 ± 0.20	2.10 ± 0.18	5.21	0.001	Significant
II	2.48 ± 0.22	2.25 ± 0.20	4.18	0.002	Significant
III	2.50 ± 0.25	2.22 ± 0.21	5.89	0.001	Significant
IV	2.47 ± 0.23	2.05 ± 0.19	4.61	<0.001	Significant

Intergroup Comparison of PCD Thickness Reduction

The mean PCD reduction differed among the four instrumentation groups. The mean reductions were 0.35 ± 0.10 mm in Group I, 0.23 ± 0.08 mm in Group II, 0.28 ± 0.11 mm in Group III, and 0.42 ± 0.09 mm in Group IV (Table 3).

**Table 3:** Intergroup comparison of mean pericervical dentin thickness reduction. One-way ANOVA: F = 7.50; p = 0.001.

Group	File system	n	Mean reduction (mm)	SD (mm)
I	Hand files (2%)	10	0.35	0.1
II	TruNatomy (4%)	10	0.23	0.08
III	ProTaper Gold (F2)	10	0.28	0.11
IV	HyFlex rotary (6%)	10	0.42	0.09

One-way ANOVA demonstrated a statistically significant difference in mean PCD reduction among the four instrumentation groups (F(3,36) = 7.50, p = 0.001). The overall analysis therefore indicated that PCD reduction differed across the instrumentation systems. The greatest numerical reduction was observed in the HyFlex group, whereas the smallest was observed in the TruNatomy group.

Post Hoc Comparison of PCD Thickness Reduction

Tukey's post hoc test was performed to identify differences in PCD thickness reduction between individual groups. According to the reported post hoc analysis, none of the pairwise comparisons reached statistical significance (p > 0.05). Although the overall analysis indicated variation among the groups, no statistically significant difference was reported between any two specific groups (Table 4).

**Table 4:** Post hoc comparison of pericervical dentin thickness reduction.

Comparison	Mean difference (I-J), mm	Standard error	p-value	Significance
Group I vs. Group II	0.12	0.045	0.142	NS
Group I vs. Group III	0.07	0.046	0.398	NS
Group I vs. Group IV	-0.07	0.047	0.402	NS
Group II vs. Group III	-0.05	0.044	0.563	NS
Group II vs. Group IV	-0.19	0.046	0.081	NS
Group III vs. Group IV	-0.14	0.045	0.178	NS

Fracture resistance

The fracture resistance values for the four instrumentation groups are presented in Table 5. The mean fracture resistance was 635.42 ± 59.87 N in Group I, 706.73 ± 134.52 N in Group II, 724.91 ± 149.38 N in Group III, and 654.68 ± 139.76 N in Group IV.

**Table 5:** Fracture resistance of the experimental groups.

Group	File system	n	Mean fracture resistance ± SD (N)	Standard error (N)	Minimum (N)	Maximum (N)
I	Hand files (2%)	10	635.42 ± 59.87	18.94	541.36	721.58
II	TruNatomy (4%)	10	706.73 ± 134.52	42.56	522.14	879.65
III	ProTaper Gold (F2)	10	724.91 ± 149.38	47.26	512.48	921.37
IV	HyFlex (6%)	10	654.68 ± 139.76	44.18	503.27	898.92

The highest numerical mean fracture resistance was observed in Group III, followed by Groups II, IV, and I. The minimum and maximum fracture resistance values were 541.36-721.58 N in Group I, 522.14-879.65 N in Group II, 512.48-921.37 N in Group III, and 503.27-898.92 N in Group IV.

One-way ANOVA was performed to determine whether fracture resistance differed significantly among the four instrumentation groups. Based on the reported group means, standard deviations, and sample sizes, the calculated ANOVA yielded F(3,36) = 1.12, p = 0.352, indicating no statistically significant difference in fracture resistance among the groups.

Because the overall ANOVA was not statistically significant, the numerical differences among the groups were interpreted descriptively rather than as evidence of statistically significant differences. Tukey's post hoc comparisons likewise did not demonstrate statistically significant pairwise differences.

Post Hoc Comparison of Fracture Resistance

Pairwise comparisons of fracture resistance are presented in Table 6. The largest mean difference was observed between Groups I and III (-89.49 N), whereas the smallest absolute difference was observed between Groups II and III (-18.18 N). According to the currently reported Tukey analysis, none of the pairwise comparisons reached statistical significance (p > 0.05) (Table 6).

**Table 6:** Post hoc comparison of fracture resistance.

Comparison	Mean difference (I-J), N	Standard error (N)	p-value	Significance
Group I vs. Group II	-71.31	55.08	0.41	NS
Group I vs. Group III	-89.49	56.36	0.3	NS
Group I vs. Group IV	-19.26	54.82	0.84	NS
Group II vs. Group III	-18.18	59.94	0.9	NS
Group II vs. Group IV	52.05	58.66	0.6	NS
Group III vs. Group IV	70.23	59.71	0.46	NS

## Discussion

Preservation of sound tooth structure is an important consideration in contemporary endodontics because the amount and distribution of remaining dentin may influence the mechanical behavior of root canal-treated teeth. The present in vitro study evaluated the effects of four instrumentation systems with different taper characteristics on PCD preservation and fracture resistance in mandibular first premolars. Pre- and post-instrumentation CBCT measurements demonstrated a reduction in PCD thickness in all four groups. The lowest numerical PCD reduction was observed with TruNatomy (0.23 ± 0.08 mm), whereas the highest numerical reduction was observed with HyFlex (0.42 ± 0.09 mm). ProTaper Gold demonstrated the highest numerical fracture resistance (724.91 ± 149.38 N), followed by TruNatomy (706.73 ± 134.52 N), HyFlex (654.68 ± 139.76 N), and hand files (635.42 ± 59.87 N). The intergroup findings should be interpreted according to the validated statistical analysis rather than on the basis of numerical differences alone.

The amount of dentin removed is a natural consequence of mechanical root canal preparation, as shown by the significant decrease in PCD thickness in all groups. Unlike the study by Shahriari S et al. [18], who reported significantly greater PCD loss in the hand-file group compared with the rotary group using ProFile instruments, the present study did not find a statistically significant difference between the hand-file and TruNatomy groups with respect to PCD loss, although the hand-file group showed greater numerical loss than the TruNatomy group. These differences could be due to variations in tooth morphology, measurement methods, instrumentation techniques, and the use of CBCT-derived PCD measurements in the current study.

The present findings can also be compared with those of Zinge PR and Patil J [19], who evaluated PCD preservation using different rotary and reciprocating single-file systems in mandibular premolars with CBCT analysis. Their findings demonstrated differences in dentin removal among the evaluated instrumentation systems. In the present study, TruNatomy demonstrated the lowest numerical PCD reduction (0.23 ± 0.08 mm), whereas HyFlex demonstrated the highest numerical reduction (0.42 ± 0.09 mm). These findings suggest that dentin removal may be influenced by factors in addition to nominal taper, including file geometry, cross-sectional design, flexibility, cutting efficiency, kinematics, and instrumentation sequence.

The relatively low numerical PCD reduction observed with TruNatomy is consistent with the conservative shaping characteristics associated with this instrumentation system. Silva EJ et al. [20] compared TruNatomy and ProTaper Gold using micro-computed tomography and reported differences in dentin removal between the systems, including greater coronal dentin removal with ProTaper Gold in specific regions. In the present study, TruNatomy also demonstrated a lower numerical PCD reduction than ProTaper Gold. However, the difference observed in the present study should be interpreted in the context of the overall statistical analysis and the differences in tooth type, measurement location, and instrumentation protocols between studies.

The instrumentation approaches involving hand files, TruNatomy, ProTaper Gold, and HyFlex EDM were evaluated by Shyma P et al. [21] in extracted mandibular premolars using pre- and post-instrumentation CBCT and fracture resistance testing, providing a particularly relevant comparison. The mean fracture resistance values for the different file systems in their study were 642.37 N, 706.67 N, 720.96 N, and 646.93 N for hand files, TruNatomy, ProTaper Gold, and HyFlex EDM files, respectively. The values for each file system in the present study were quite similar, with mean fracture resistance values of 635.42 N, 706.73 N, 724.91 N, and 654.68 N for hand files, TruNatomy, ProTaper Gold, and HyFlex EDM files, respectively. The fracture resistance values observed in the present study were numerically similar to those reported by Shyma P et al. [21]. However, neither study demonstrated a statistically significant difference among the evaluated instrumentation systems. Therefore, the numerical differences observed in fracture resistance should be interpreted descriptively rather than as evidence of superior performance.

The differences from the present results could be attributed to the widely varying instrumentation philosophies and experimental designs used. Self-adjusting file (SAF) is an adaptive hollow-file system that removes dentin circumferentially rather than through traditional rotary cutting, whereas the present study involved fixed-, progressive-, and regressive-taper systems.

An important observation in the present study was that the group with the lowest numerical PCD reduction did not demonstrate the highest numerical fracture resistance. TruNatomy showed the lowest numerical PCD reduction, whereas ProTaper Gold demonstrated the highest numerical fracture resistance. This finding indicates that fracture resistance cannot be interpreted solely on the basis of the amount of dentin removed. The mechanical behavior of endodontically treated teeth may also be influenced by the geometry and distribution of remaining dentin, canal configuration, preparation characteristics, root morphology, restorative procedures, and loading conditions. Therefore, the relationship between dentin preservation and fracture resistance should be interpreted as multifactorial rather than being attributed to file taper alone.

ProTaper Gold demonstrated the highest numerical fracture resistance in the present study (724.91 ± 149.38 N). This finding may be considered in relation to the mechanical and metallurgical characteristics of heat-treated NiTi instruments; however, the present study was not designed to determine whether these characteristics directly accounted for the observed fracture resistance. Therefore, the higher numerical value observed with ProTaper Gold should be interpreted descriptively and not attributed to a single instrument property.

Although the overall ANOVA indicated a statistically significant difference in PCD reduction among the groups, Tukey's post hoc analysis did not identify statistically significant differences between individual groups. Therefore, the findings should be interpreted as evidence of an overall group effect without statistically significant pairwise differences.

The results obtained for fracture resistance are particularly important when considered alongside the PCD results. The TruNatomy group had the lowest reported mean dentin loss, indicating a conservative shaping profile. ProTaper Gold, however, demonstrated slightly higher fracture resistance with greater reported PCD reduction. It is important to note that this apparent contradiction highlights that the amount of remaining dentin is not the only factor influencing fracture behavior. Instead, the distribution and continuity of the remaining dentin might be more important in determining how stresses are transmitted through the tooth.

Limitations

This study has certain limitations, including its in vitro design, relatively small sample size (10 teeth per group), and inability to replicate physiological periodontal ligament support and clinical occlusal loading conditions. Fracture resistance was assessed under static loading, which may not reflect the multidirectional and cyclic forces encountered clinically [22,23]. Additionally, different obturation techniques were used across groups, potentially influencing stress distribution and fracture resistance. Future studies should employ larger sample sizes, standardized obturation protocols, and dynamic loading models to improve clinical relevance.

## Conclusions

All four instrumentation systems produced measurable reductions in PCD thickness following root canal preparation. TruNatomy demonstrated the lowest numerical PCD reduction, whereas HyFlex demonstrated the highest numerical reduction. ProTaper Gold showed the highest numerical fracture resistance, followed by TruNatomy, HyFlex, and hand files. Overall, the findings suggest that file taper alone does not determine PCD preservation or fracture resistance.
